# Efficacy of high-dose serrapeptase (serratiopeptidase) in inflammation among orthopedic trauma patients

**DOI:** 10.25122/jml-2026-0044

**Published:** 2026-07

**Authors:** Mian Muhammad Hanif, Hafiz Muhammad Shiraz, Nusrat Rasheed, Muhammad Jamil, Akhtar Hussain, Husnain Khalid, Mazhar Mahmood, Arslan Arshad, Asif Saeed, Asif Nawaz, Asad Ullah Jan, Asif Imran, Khalil Ahmed Gil, Hussain Bux, Tariq Aziz, Saad Riaz, Asghar Khan, Haider Khalid, Dilshad Hussain, Muhammad Athar Khan, Neeta Maheshwary

**Affiliations:** 1 Lahore General Hospital, Lahore, Pakistan; 2 Dow International Medical College, Dow University of Health Sciences, Karachi, Pakistan; 3 Timergara Teaching Hospital, Timergara, Pakistan; 4 Mercy Teaching Hospital, Peshawar, Pakistan; 5 District Head Quarter Hospital, Faisalabad, Pakistan; 6 Sialkot Medical College, Sialkot, Pakistan; 7 Hayatabad Medical Complex, Peshawar, Pakistan; 8 Mardan Medical College, Mardan, Pakistan; 9 Razia Saeed Hospital, Multan, Pakistan; 10 Sir Syed College of Medical Sciences for Girls, Karachi, Pakistan; 11 District Head Quarter Hospital, Swat, Pakistan; 12 Rawalpindi Teaching Hospital, Rawalpindi, Pakistan; 13 Saidu Teaching Hospital, Saidu Sharif, Swat, Pakistan; 14 Medical Affairs Department, Helix Pharma Pvt Ltd, Karachi, Pakistan; 15 Department of Community Medicine, Liaquat College of Medicine & Dentistry, Karachi, Pakistan

**Keywords:** serratiopeptidase, serrapeptase, orthopedic trauma, anti-inflammatory agents, proteolytic enzymes, orthopedics, wounds and injuries

## Abstract

Pain and inflammation resulting from orthopedic trauma have a significant negative impact on early recovery and rehabilitation. A proteolytic enzyme called serrapeptase (serratiopeptidase), which has anti-inflammatory properties, has been used with increasing frequency in surgery and trauma-related surgery. Nevertheless, clinical evidence of its efficacy in orthopedic trauma remains scant. The objective of the study was to determine the effectiveness, safety, and tolerability of high-dose serrapeptase (serratiopeptidase; DANZ-N 30 mg) used as an adjunctive therapy in reducing inflammation and pain in patients with orthopedic trauma. This was a prospective, multicenter, real-world observational study that enrolled 850 patients with orthopedic trauma injuries. Group assignment was at the discretion of the treating physician: patients received either conventional trauma care alone or conventional trauma care combined with oral serrapeptase. Pain intensity was assessed using a Visual Analog Scale (VAS), and inflammatory changes were systematically monitored over 15 days. Adverse events were systematically recorded to assess safety. In total, 408 patients in the serrapeptase group and 405 in the conventional-treatment group completed the study. VAS scores declined significantly faster in the serrapeptase group, with a mean difference of 1.4 points by day 15 (*P* < 0.001). All inflammatory signs resolved more rapidly with serrapeptase (*P* < 0.001 across all parameters by day 15). Adverse events were mild and comparable between groups, with no serious events reported. Adjunctive serrapeptase (serratiopeptidase; DANZ-N 30 mg) was associated with lower pain scores and fewer clinical signs of inflammation in orthopedic trauma patients.

## Introduction

Inflammation is a key pathophysiological mechanism of response to traumatic injuries and surgical operations. Although it plays a protective role at the initial stages of the wound healing process, a persistent or excessive inflammatory response plays a significant role in tissue damage, pain, edema, and delayed recovery. Post-trauma inflammation management is therefore a vital part of early rehabilitation of surgical and orthopedic patients [1,2]; however, the use of non-steroidal anti-inflammatory drugs (NSAIDs) and corticosteroids has significant side effects, including gastrointestinal injury, renal dysfunction, immunosuppression, and disturbance of the wound-healing cascade [3,4].

Herein, enzyme-based anti-inflammatory drugs have come in as a good substitute. Of these, a zinc-dependent metalloprotease called serratiopeptidase (also known as serrapeptase) obtained as a derivative of silkworm E-15, *Serratia marcescens* E-15, has been noted to have a wide anti-inflammatory and fibrinolytic effect [5] with proteolytic properties to hydrolyze inflammatory mediators (e.g., bradykinin, histamine, serotonin) and reduce capillary permeability and edema [2,3]. Serratiopeptidase also possesses analgesic, mucolytic, and antiedematous activity and has been used in dental, orthopedic, gynecologic, and ear, nose, and throat (ENT) practices [6].

In pharmacokinetics, the bioavailability of serratiopeptidase oral delivery is systemic in nature, especially when it is administered in enteric-like coatings. It is not metabolized on its way to the active site but gets absorbed intact directly through the gastrointestinal route, possibly by clathrin-mediated endocytosis, to reach its peak concentrations in inflamed tissues in 60 to 90 minutes [7] and does not inhibit the formation of prostaglandins (the cyclooxygenase [COX] pathway), and, therefore, maintains physiological healing and hemostatic balance [8].

A particularly valued property of serratiopeptidase is its ability to break down and dissolve necrotic or damaged tissue while sparing healthy tissue. This selective action helps reduce inflammatory swelling (edema) more effectively and gently than traditional anti-inflammatory agents [9]. Similar reductions in pain and swelling have been documented in clinical settings following single-visit endodontic treatment, where serratiopeptidase showed superior anti-edema effects compared with the NSAID ibuprofen [10].

Nevertheless, there remains a lack of robust, large-scale, trauma-focused trials assessing the use of high-dose serratiopeptidase as a monotherapy. Many existing studies utilize fixed-dose combinations (FDCs) with bromelain, rutoside, and trypsin—agents known to have a very promising role in relieving inflammation and promoting wound healing [11]. Furthermore, inconsistent dosing regimens and outcome measures have hampered meta-analytic assessment. Most published trials have used doses ranging from 10–30 mg twice daily; however, some experimental designs have proposed higher doses for more extensive tissue trauma [12,13].

Given the need for a safer anti-inflammatory alternative in polytrauma and surgical recovery, particularly for patients at risk for NSAID-induced gastrointestinal or renal complications, high-dose serratiopeptidase offers a mechanistically novel and potentially superior profile. Its ability to facilitate fibrinolysis, enhance microcirculation, and reduce leukocyte recruitment could theoretically shorten recovery time, minimize analgesic requirements, and reduce the incidence of post-traumatic fibrosis [14].

There is a lack of local data regarding the efficacy of serratiopeptidase in patients with inflammation in a real-world setting in the Pakistani population. The current study aimed to address a glaring gap in the evidence literature about pharmacologic treatment of trauma, as well as to outline enzyme-based treatment as a viable auxiliary to modern surgical management. The objective of this study was to assess the efficacy, safety, and tolerability of serratiopeptidase in patients with inflammation in orthopedic trauma conditions, with particular attention to pain relief, swelling reduction, and functional recovery.

## Material and Methods

The research design was a prospective, multicenter, real-world, comparative observational design conducted across 15 orthopedic and trauma centers in Pakistan. Autonomous therapeutic decision-making was practiced by clinicians without any randomization or blinding regimes. Data were acquired through standardized clinical reporting forms at five time points during a 15-day observation period.

### Participants

A total of 850 participants aged 18 to 85 years were recruited from 15 orthopedic and trauma centers in Pakistan. Eligible participants were male or female ambulatory patients with post-traumatic inflammation supported by clinical history, physical examination, and radiological evidence. Patients were required to provide written informed consent and to be willing and able to comply with all study requirements and instructions provided by the site study staff. Patients who had received any investigational drug within 30 days before Visit 1 were not eligible for inclusion. Patients were excluded if they were pregnant or lactating, or if women of childbearing potential were not using effective contraception. Additional exclusion criteria included known hypersensitivity to serratiopeptidase or paracetamol, immunocompromised status or use of systemic immunosuppressive therapy, coagulation abnormalities despite treatment, severely impaired central nervous system function, secondary osteoarthritis of the knee, articular inflammatory disease, previous surgery including arthroscopy, or planned administration of any investigational drug other than the protocol product during the study period.

### Treatment cohorts

Participants were categorized into two observational cohorts according to the treatment regimen selected by the treating physician. Cohort A included patients who received oral serratiopeptidase (DANZ-N 30 mg once daily) as an adjunct to conventional trauma care. Cohort B included patients who received conventional trauma care alone without serratiopeptidase or other enzymatic agents. Conventional trauma care consisted of paracetamol 500–1000 mg up to three times daily, rest, limb elevation, cold compresses, immobilization where clinically indicated, and physiotherapy according to injury type and clinical condition. Surgical wound care, fracture stabilization, or other trauma-specific management was provided according to routine orthopedic practice. NSAIDs, corticosteroids, and other enzymatic anti-inflammatory agents were not permitted during the 15-day observation period to reduce pharmacologic confounding of pain and inflammatory outcomes.

### Clinical assessment

**Figure 1:**
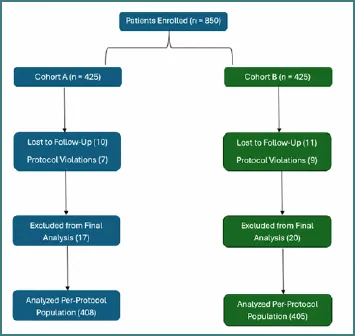
Study flow diagram

Clinical inflammatory signs were assessed using the five classical cardinal signs of inflammation: redness (rubor), swelling (tumor), local heat (calor), pain (dolor), and loss of function (functio laesa) [15]. These signs are widely recognized clinical manifestations of acute inflammation. Each inflammatory sign was recorded as present or absent. Each sign was documented as present or absent by the treating orthopedic clinician at Day 0, Day 3, Day 5, Day 7, and Day 15 using a standardized clinical reporting form. At baseline and during clinical indications (Day 15), radiographic imaging was taken. Inflammatory signs were assessed by the treating orthopedic/trauma clinician at each participating center. Inflammatory markers such as erythrocyte sedimentation rate (ESR) and C-reactive protein (CRP) were measured when necessary through laboratory investigations. Adverse events (AEs), as well as serious adverse events (SAEs), were also documented during the observation period. The patient disposition flowchart demonstrates that 17 patients were excluded because of loss to follow-up or protocol deviations in Cohort A and 20 patients were excluded in Cohort B (Figure 1).

To evaluate consistency of clinical assessment, inter-rater reliability was assessed in a subset of cases by two orthopedic/trauma clinicians using the same standardized criteria. Agreement was evaluated using Cohen’s kappa coefficient for categorical present/absent assessments. Radiographic imaging was performed at baseline and repeated when clinically indicated. Laboratory inflammatory markers, including erythrocyte sedimentation rate (ESR) and C-reactive protein (CRP), were obtained when considered clinically necessary, but were not measured systematically in all participants and were therefore not used as primary outcome measures.

The primary endpoints of the study included the change in VAS pain scores and resolution of the clinical manifestations of inflammation between the baseline and Day 15. Secondary endpoints were based on the incidence and severity of AEs and SAEs, and any treatment discontinuations that could be due to intolerance. The sample size was calculated using data presented by Tamimi *et al*. [12], where the researchers reported a moderate effect size (Cohen’s d = 0.43) in reducing edema after the application of serratiopeptidase therapy. To achieve a two-sided alpha of 0.05 and power of 90, the participants calculated per group should be at least 113. However, a total of 850 participants were sought to increase generalizability, provide subcohort analysis, and address the intercenter variability.

### Statistical analysis

Statistical analysis was performed using SPSS version 25. Continuous variables were assessed for normality using the Shapiro–Wilk test. Normally distributed continuous variables were presented as mean ± standard deviation and compared between groups using the independent samples *t*-test. Categorical variables were summarized as frequencies and percentages and compared using the chi-square test. Changes in VAS pain scores across the five assessment points were analyzed using an independent *t*-test. Since five clinical inflammatory signs were assessed at each follow-up time point, the Bonferroni-adjusted significance threshold was set at *P* < 0.01 for these comparisons. For primary outcomes, a two-sided *P* value < 0.05 was considered statistically significant.

## Results

A total of 850 patients were enrolled from 15 orthopedic trauma centers. At enrollment, 425 patients received oral serratiopeptidase in addition to conventional trauma care, while 425 patients received conventional care alone. After excluding patients who were lost to follow-up or had protocol deviations, 408 patients in Cohort A and 405 patients in Cohort B were included in the final per-protocol analysis. Inter-rater reliability for clinician-recorded inflammatory signs showed almost perfect agreement, with a Cohen’s kappa value of 0.821. This supported consistency in the clinical assessment of redness, swelling, local heat, pain/tenderness, and loss of function.

The two cohorts were generally comparable at baseline (Table 1). The mean age was similar in Cohort A and Cohort B (38.2 ± 12.1 vs. 37.9 ± 11.5 years), and there was no significant difference in sex distribution between the groups. The pattern of trauma was also similar, with comparable numbers of soft-tissue injuries and surgically treated trauma cases in both cohorts (all *P* >0.05).

**Table 1:** Baseline characteristics of the study population

Variable	Cohort A (serratiopeptidase) (*n* = 408)	Cohort B (control) (*n* = 405)	*P* value
Age (mean ± SD)	38.2 ± 12.1	37.9 ± 11.5	0.64
Male, *n* (%)	234 (57.4%)	219 (54.1%)	0.38
Female, *n* (%)	174 (42.6%)	186 (45.9%)	
Soft tissue injuries	144	144	0.99
Surgical trauma cases	120	117	0.61

Data are presented as mean ± SD or *n* (%). *P* values from independent-samples *t*-test and chi-square test.

Mean VAS pain scores were similar between the two groups at baseline (7.8 ± 1.0 in Cohort A vs. 7.9 ± 1.1 in Cohort B; *P* = 0.18). From Day 3 onward, Cohort A had significantly lower VAS scores than Cohort B at each follow-up visit. The between-group mean difference was 1.3 points on Day 3, equivalent to approximately 13 mm on a 100-mm VAS (95% CI, 1.11–1.49; *P* < 0.001), and remained 1.3–1.4 points through Day 15. By Day 15, the mean VAS score was 1.5 ± 0.9 in Cohort A compared with 2.9 ± 1.0 in Cohort B, with a mean difference of 1.4 points, equivalent to approximately 14 mm on a 100-mm VAS (95% CI, 1.27–1.53; *P* < 0.001) (Table 2).

**Table 2:** Changes in VAS pain scores over time (Mean ± SD)

Timepoint	Cohort A (serratiopeptidase) (mean ± SD)	Cohort B (control) (mean ± SD)	Mean difference (B-A)	95% CI for difference	*P* value
Day 0	7.8 ± 1.0	7.9 ± 1.1	0.1	-0.04 to 0.24	0.18
Day 3	5.1 ± 1.3	6.4 ± 1.5	1.3	1.11 to 1.49	<0.001
Day 5	3.6 ± 1.2	4.9 ± 1.4	1.3	1.12 to 1.48	<0.001
Day 7	2.4 ± 1.1	3.8 ± 1.3	1.4	1.23 to 1.57	<0.001
Day 15	1.5 ± 0.9	2.9 ± 1.0	1.4	1.27 to 1.53	<0.001

Data are presented as mean ± SD. Comparisons between cohorts were made using the independent-samples *t* test; a Bonferroni-adjusted significance threshold of *P* < .01 was applied. Abbreviations: CI, confidence interval; SD, standard deviation; VAS, Visual Analog Scale.

Clinical inflammatory signs were recorded at Day 0, Day 3, Day 5, Day 7, and Day 15 (Table 3). At baseline, the proportions of patients with redness, swelling, local heat, pain, and loss of function were comparable between the two groups. By Day 3, swelling was significantly less frequent in Cohort A than in Cohort B after Bonferroni adjustment, while the remaining signs showed favorable trends. From Day 5 onward, all five inflammatory signs were significantly less frequent in Cohort A compared with Cohort B. By Day 15, Cohort A continued to show lower frequencies of redness, swelling, heat, pain, and loss of function.

**Table 3:** Comparison of clinical inflammatory signs over time

Day	Sign	Cohort A *n* (%)	Cohort B *n* (%)	*P* value
Day 0	Redness	265 (65%)	267 (66%)	> 0.05
Day 0	Swelling	286 (70%)	288 (71%)	> 0.05
Day 0	Heat	253 (62%)	255 (63%)	> 0.05
Day 0	Pain	359 (88%)	360 (89%)	> 0.05
Day 0	Loss of function	306 (75%)	308 (76%)	> 0.05
Day 3	Redness	204 (50%)	235 (58%)	0.026
Day 3	Swelling	224 (55%)	263 (65%)	0.004
Day 3	Heat	196 (48%)	231 (57%)	0.012
Day 3	Pain	294 (72%)	324 (80%)	0.010
Day 3	Loss of function	245 (60%)	275 (68%)	0.023
Day 5	Redness	163 (40%)	203 (50%)	0.004
Day 5	Swelling	184 (45%)	243 (60%)	<0.001
Day 5	Heat	143 (35%)	187 (46%)	<0.001
Day 5	Pain	237 (58%)	284 (70%)	<0.001
Day 5	Loss of function	204 (50%)	247 (61%)	<0.001
Day 7	Redness	102 (25%)	182 (45%)	<0.001
Day 7	Swelling	122 (30%)	211 (52%)	<0.001
Day 7	Heat	82 (20%)	166 (41%)	<0.001
Day 7	Pain	163 (40%)	243 (60%)	<0.001
Day 7	Loss of function	143 (35%)	223 (55%)	<0.001
Day 15	Redness	49 (12%)	101 (25%)	<0.001
Day 15	Swelling	73 (18%)	130 (32%)	<0.001
Day 15	Heat	37 (9%)	81 (20%)	<0.001
Day 15	Pain	90 (22%)	146 (36%)	<0.001
Day 15	Loss of function	61 (15%)	113 (28%)	<0.001

Data are presented as *n* (%). Comparisons between cohorts were made using the χ^2^ test with Yates' continuity correction; a Bonferroni-adjusted significance threshold of *P* < .01 was applied. Cohort A, serratiopeptidase; Cohort B, control.

**Figure 2:**
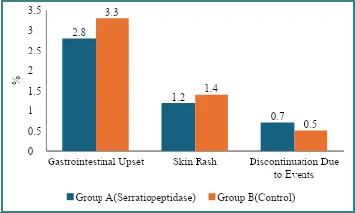
Adverse events during the study period. Gastrointestinal upset, *P* = 0.65; skin rash, *P* = 0.77; discontinuation due to events, *P* = 0.89 (χ^2^ test).

Adverse events were uncommon and comparable between the two groups (Figure 2). Gastrointestinal upset occurred in 12 (2.8%) of patients in Cohort A and 14 (3.31%) in Cohort B (*P* = 0.65), while skin rash was reported in 1.2% and 1.4%, respectively (*P* = 0.77). Discontinuation due to adverse events was rare in both groups (0.7% vs. 0.5%; *P* = 0.89). Overall, no statistically significant differences in adverse events were observed, indicating good tolerability of serratiopeptidase as adjunctive therapy.

## Discussion

The extensive, ongoing, descriptive, real-life study proves that serratiopeptidase, used as an adjunct to standard care in orthopedic trauma, hastens pain reduction, reduces inflammation, and supports functional recovery without increasing the number of adverse events. The findings align with previously reported pharmacological and clinical trials and provide new data from a multicenter study reflective of real-life therapeutic practice.

The VAS pain score reductions observed from Day 3 onward in our Cohort A are both statistically and clinically significant. The difference exceeded the minimum clinically important difference (MCID) of 1.3 points as defined for musculoskeletal pain. This aligns with findings from Tamimi *et al*., who showed a mean VAS reduction of 3.9 mm with serrapeptase vs placebo after oral surgery [12]. Krishna *et al.’s* study also demonstrated its analgesic and anti-inflammatory effects after impacted third molar removal [16].

Besides analgesia, the reduction of inflammatory markers, swelling, erythema, and functional impairment had a faster and longer-lasting effect in the serrapeptase cohort. Improvement in objective signs was exhibited by day 15 in more than 85% of respondents, versus 72 % in the control cohort. These results are in line with other studies conducted before by Bhagat *et al.*, as they reported a decrease in infiltration, exudate viscosity, and tissue congestion with enzymatic therapy [17]. Sakurada *et al*. found serrapeptase to be a potent inhibitor of interleukin-6 (IL-6) and tumor necrosis factor-alpha (TNF-α) levels and thus identified the immunomodulatory effect of serrapeptase [18].

We also find support from Jadav *et al*., who found that serrapeptase showed better joint performance and faster rates of swelling reduction compared to diclofenac in orthopedic patients [19]. Satamati *et al*., likewise reported improved mobility and faster reduction in ankle joint edema in trauma patients on serrapeptase [20]. These improvements are not solely symptomatic; they likely reflect reduced fibrin deposition and improved lymphatic drainage, key mechanisms supported by Jadhav *et al*. in proteomic studies of enzyme action [21]. Garg *et al*. evaluated and compared the efficacy and safety of serrapeptase and aceclofenac in reducing swelling and pain following traumatic soft tissue injury. Serrapeptase showed significant anti-inflammatory effect with mild analgesic action. Serrapeptase can be used as an anti-inflammatory agent in traumatic bony and soft tissue injuries in the oral and maxillofacial region [9].

From a safety perspective, the current study aligns with previous large-scale evaluations demonstrating low adverse event rates with serrapeptase. Bapat *et al*. confirmed the effectiveness and tolerability of a fixed-dose combination (FDC) of serratiopeptidase and diclofenac sodium in postoperative obstetric and gynecologic (OB/GYN) surgery patients during the early postoperative period [22]. Our data, showing less than 1% discontinuation due to adverse events, confirm the excellent tolerability profile of serrapeptase, particularly compared with non-selective NSAIDs and corticosteroids, which are often associated with gastrointestinal, renal, or cardiovascular adverse effects. Previous studies have also demonstrated potential therapeutic applications of serrapeptase—alone or in combination with other agents—across a range of conditions including rheumatoid arthritis, atherosclerosis, amyloidosis, sinusitis, bronchitis, and carpal tunnel syndrome [23-27].

Although the prospective multicenter design reflects routine clinical practice, the observational nature of the study is an important limitation. Treatment allocation was not randomized or blinded and was directed by the treating physician. This may have introduced selection bias, performance bias, assessment bias, and confounding by indication. Therefore, the observed lower pain scores and fewer inflammatory signs among patients receiving adjunctive serratiopeptidase should be interpreted as associations rather than evidence of a causal treatment effect. Further randomized and controlled studies are needed, including studies in pediatric populations, to confirm the safety and clinical utility of serratiopeptidase across different age groups. Although NSAIDs, corticosteroids, and other enzymatic anti-inflammatory agents were not permitted, other supportive measures such as immobilization, physiotherapy, wound care, and fracture stabilization were provided according to clinical need and physician judgment. Therefore, variation in co-interventions may have influenced pain and inflammatory outcomes.

## Conclusion

Adjunctive serrapeptase (serratiopeptidase; DANZ-N 30 mg) use was associated with lower pain scores and fewer clinician-recorded inflammatory signs during follow-up in this real-world orthopedic trauma cohort. However, because the study was non-randomized and non-blinded, with physician-directed treatment allocation, the findings should be interpreted as associative and hypothesis-generating rather than causal. These results support the need for randomized, blinded controlled trials to evaluate further the clinical role of serrapeptase in orthopedic trauma care.

## Data Availability

Data are available upon reasonable request.

## Key findings

In this multicenter, prospective observational study, serratiopeptidase added to standard care significantly reduced pain and inflammatory signs compared with conventional treatment alone. It provides real-world evidence for the efficacy, safety, and tolerability of serratiopeptidase in orthopedic trauma, with improvements across fracture, soft-tissue, and post-surgical subgroups.

## Implications for practice

The findings support serratiopeptidase as an adjunct in multimodal trauma care and may reduce reliance on conventional anti-inflammatory drugs and their associated side effects. They may also inform future clinical guidelines and encourage randomized trials comparing enzyme therapy with NSAIDs.
